# Strong stimulation of recombinant protein production in *Escherichia coli *by combining stimulatory control elements in an expression cassette

**DOI:** 10.1186/1475-2859-11-133

**Published:** 2012-10-02

**Authors:** Friederike Zwick, Rahmi Lale, Svein Valla

**Affiliations:** 1 Department of Biotechnology, Norwegian University of Science and Technology, Sem Sælands Vei 6/8, N-7491, Trondheim, Norway

**Keywords:** Recombinant, XylS/Pm, UTR, Gene expression, Promoter, Mutant, Escherichia coli

## Abstract

**Background:**

The XylS/*Pm* expression system has been used to produce recombinant proteins at industrial levels in *Escherichia coli*. Activation of transcription from the *Pm* promoter takes place in the presence of benzoic acid or derivatives of it. Previous mutagenesis studies resulted in identification of several variants of the expression control elements *xylS* (X), *Pm* (P) and the 5'-untranslated region (U) that individually gave rise to strongly stimulated expression. The goal of this study was to test if combination of such stimulatory mutations in the same expression vectors would lead to further increase of expression levels.

**Results:**

We combined X, P and U variants that were originally identified due to their ability to strongly stimulate expression of the reporter gene *bla* (resistance to penicillin). Combination of optimized elements stimulated *bla* expression up to 75-fold (X, P and U combined) relative to the wild-type system, while accumulated transcript levels increased about 50-fold. This is much more than for the elements individually. We also tested combination of the variant elements on two other and unrelated genes, *celB* (encoding phosphoglucomutase) and the human growth factor gene *gm-csf*. Protein production from these genes is much more efficient than from *bla* in the wild-type system, but expression was still significantly stimulated by the combination of X, P and U variants, although not to the same extent as for *bla*.

We also integrated a single copy of the expression cassette with each gene into the *E. coli* chromosome and found that the expression level from this single copy was higher for *bla* than for the wild-type plasmid system, while it was lower for *celB* and *gm-csf*.

**Conclusion:**

Our results show that combination of stimulatory expression control elements can be used to further increase production of different proteins in *E. coli*. For one reporter gene (*bla*) this allowed for more protein production from a single gene copy integrated on the chromosome, compared to the wild-type plasmid system. The approach described here should in principle be applicable for improvement of any expression cassette.

## Background

Plasmids are heavily utilized as expression tools for recombinant protein production in bacteria, mainly because they enable easy introduction of recombinant genes at high gene dosages, resulting in large amounts of transcripts from the gene of interest [1]. However, high plasmid copy numbers in itself represent a burden for the host [2-4], and this may lead to problems like plasmid instability, decreased growth rates, and plasmid DNA mutations [2,5-8]. Antibiotic selection markers included on plasmids for enhanced stability also impose an additional metabolic burden to the cell [9].

Ideally it would therefore be advantageous to avoid such autonomously replicating elements and chromosomal integration of expression cassettes stands as an attractive alternative for stable expression [10,11].

The main problem with single copy chromosomal integrations is that the strong reduction in gene dosage compared to high copy number plasmids leads to reduced transcript formation and will therefore require very efficient translation of each transcript. Increasing the number of copies of the gene of interest on the chromosome could potentially eliminate this problem, but this requires further modification of the host genome in order to ensure the stability of the multiple times integrated DNA [11]. It is therefore important to develop methods that aim for the highest possible levels of expression per gene copy, as this might allow reduction of the number of gene copies per cell. A recent study demonstrates an approach to ensure high levels of transcript formation, by placing the desired gene together with a tandem *tac* promoter cluster into the chromosome [12]. This method eliminates the problem of having multiple copies of the gene, however the promoter utilized is constitutive. For metabolic engineering type applications it is desirable to have time-dependent expression.

We have previously demonstrated that the strong and positively regulated *xylS/Pm* expression cassette in its wild-type form, combined with a mini-RK2 replicon, can serve as a tool to achieve industrial level production of recombinant proteins in *Escherichia coli*[13,14]. XylS belongs to the AraC-XylS transcriptional regulator family and in the presence of passively transported benzoic acid derivates it activates transcription from the *Pm* promoter.

More recently, we have shown that expression from *Pm* can be further strongly stimulated by introducing mutations in the promoter region [15], in the 5'-untranslated region of mRNA (5'-UTR) [16], or in the XylS regulator coding sequence [17]. In this study we show that combination of such previously identified variant DNA control elements leads to additive stimulatory effects on expression, and this approach should in principle be applicable to any expression system.

## Results

### The expression level from *Pm* can be strongly stimulated by combining mutated DNA elements previously shown to individually enhance expression

**Figure 1:**
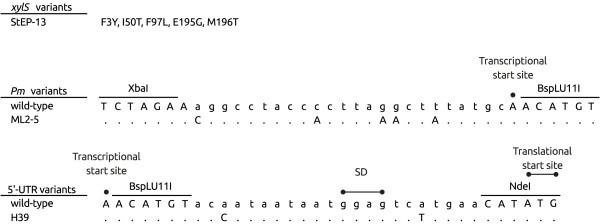
**Sequence profiles of the variants that are used in this study. **Restriction sites shown are unique. Nucleotides in lower case indicate randomly mutagenized bases, and introduced mutations are indicated for each construct (amino acid substitutions for XylS, and base changes for *Pm* and 5'-UTR variants). Identical nucleotides are indicated by dots, deletion mutations are indicated by short horizontal lines. Transcriptional and translational start sites are written in bold face and indicated with filled circles. SD is the putative Shine-Dalgarno sequence.

Initial studies involving combinations of previously isolated stimulatory control element variants (*Pm* promoter, its UTR and *xylS*) indicated that they at least to some extent acted additively, and based on these observations cells containing eight different plasmids were subjected to more detailed analyses. A plasmid with only wild-type elements was used as control, and in addition we constructed three plasmids containing one variant element only (comX, comP, comU), three constructs with each possible combination of two variant elements (comXP, comXU, comPU), and one construct (comXPU) in which all three elements were combined (Table 1). The three variants used were the *xylS* variant StEP-13 (X), which carries five amino acid substitutions and stimulates transcription [17]; a promoter variant (P) designated ML2-5 which carries five point mutations in *Pm*, also stimulating transcription [15]; and a 5'-UTR variant (U) designated H39 with two point mutations (Figure 1), which appears to predominantly stimulate translation.

**Table 1:** **Expression profiles of *E****.****coli *DH5α cells containing the indicated constructs**

**Combinations**	***xylS***	***Pm***	**5'-UTR**	Induced*	Uninduced*	**Reference**
wild-type	wild-type	wild-type	wild-type	0.2 - 0.4	0.005 - 0.01	This study
comP	wild-type	ML2-5	wild-type	1 – 2	0.005 - 0.01	[15]
comU	wild-type	wild-type	H39	2 – 3	0.06 - 0.08	This study
comX	StEP-13	wild-type	wild-type	1 – 2	0.02 - 0.03	[17]
comPU	wild-type	ML2-5	H39	5 – 6	1 - 1.5	This study
comXP	StEP-13	ML2-5	wild-type	3 – 4	0.3 - 0.4	This study
comXU	StEP-13	wild-type	H39	12 – 13	0.8 - 0.9	This study
comXPU	StEP-13	ML2-5	H39	15 – 20	3 - 4	This study

* Phenotypes are given as ampicillin resistance levels (mg mL^-1^) under induced (0.1 mM m-toluate) and uninduced conditions.

**Figure 2:**
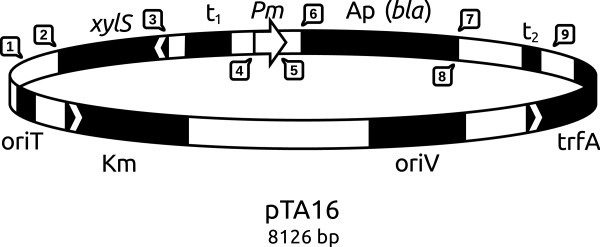
**Map of plasmid pTA16.***Pm, *positively regulated promoter; *xylS, *gene encoding *Pm *activator; Ap^r^*(bla), *ampicillin resistance gene encoding β*-*lactamase; Km^r^, kanamycin resistance gene; *trfA, *gene encoding the essential replication protein; *oriV*, origin of vegetative replication; *oriT, *origin of conjugal transfer; *t1, rrnBTlT2* bidirectional transcriptional terminator; *t2,* bidirectional transcriptional terminator. Restriction sites shown are unique: 1-Sfil, 2-Agel, 3-Sacl, 4-Xbal, 5-BspLUlll, 6-Ndel, 7-Notl, 8-BamH1, 9-Kpn1.

To monitor the expression from the different variants and the combination of them we first used *bla* (encoding β-lactamase) as a reporter gene. This gene was also used as a reporter to originally identify the variant sequences, based on the observation that host tolerance against ampicillin correlates with the amounts of β-lactamase produced [15-17]. The *bla* gene was in all cases expressed from the relevant variant version of the plasmid pTA16 (Figure 2).

The expression level from *Pm* can be continuously adjusted by varying the inducer concentration [18-20], and in the tests of the different combination constructs we used a low concentration (0.1 mM) to avoid potential host toxicity effects caused by elevated expression levels of β-lactamase. Plating of the host strains containing the plasmid variants on agar medium supplied with varying concentrations of ampicillin demonstrated that under induced conditions cells containing the wild-type, comX, comP and comU plasmid constructs tolerated up to 0.2, 1, 1, and 2 mg mL^-1^ of the antibiotic, respectively (Table 1). Interestingly, all three combinations of the two variant elements, comXP, comXU and comPU, resulted in varying but further enhancement of target protein expression, observed as upper tolerance levels of 3, 12, and 5 mg mL^-1^ ampicillin, respectively. The effect was even more drastic for comXPU, which tolerated up to 15 mg mL^-1^ of ampicillin. This is 75-fold higher than for cells with the wild-type plasmid. The uninduced resistance levels also went up for all strains (up to 3 mg mL^-1^ for comXPU).

**Figure 3:**
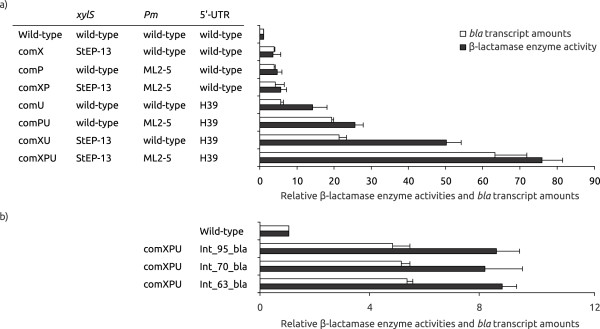
**β**-**lactamase enzyme activities and *bla *transcript amounts for extra**-**chromosomally (a) and chromosomally (b) expressed combination constructs. **Enzyme activities in white, transcript amounts in black, all values are relative to the wild-type (arbitrarily set to 1). The values are the average of at least two biological replicas. *E. coli *DH5α was used as host.

Ampicillin resistance is a good indicator of expression at the protein level, but cannot be used for accurate quantitative comparisons between clones. We therefore directly measured the corresponding β-lactamase enzyme activities. Combination of two elements in all cases stimulated expression at the protein level, about 6-fold for comXP, 25-fold for comPU, 50-fold for comXU and 75-fold for comXPU (Figure 3a). These data are in good agreement with the observed stimulation of ampicillin resistance.

The levels of accumulated transcripts of the target gene were also measured by relative quantification real-time RT-PCR (qRT-PCR), and as expected a strong stimulation was observed (up to about 63-fold for comXPU), although somewhat less than at the protein level (Figure 3a). This presumably reflects that some of the stimulation is resulting from improved translation of the target gene, most clearly demonstrated by comXU.

### Combination of variant elements also leads to increased expression of two other tested reporter genes

**Figure 4:**
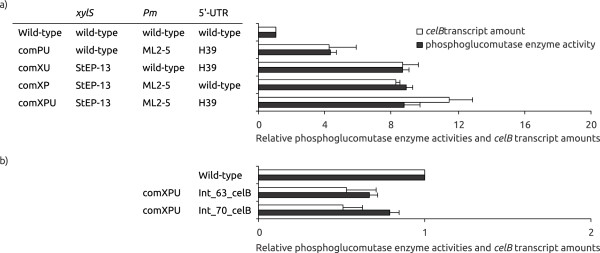
**Phosphoglucomutase enzyme activities and *celB *transcript amounts for extra**-**chromosomally (a) and chromosomally (b) expressed combination constructs.** Enzyme activities in white*,* transcript amounts in black, all values are relative to the wild-type (arbitrarily set to 1). The values are the average of at least two biological replicas. *E. coli* DH5α was used as host.

**Figure 5:**
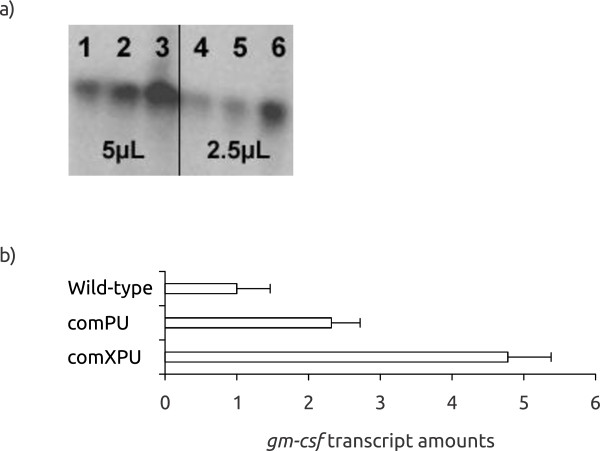
**Western Blot (a) and *gm****-****csf *transcript amounts (b) for extra**-**chromosomally expressed combination constructs.** (a) 1 and 4: wt plasmid with elevated copy number (3-4x); 2 and 5: comPU; 3 and 6 : comXPU; total cell protein was loaded on the gel (b) values are relative to the wild-type plasmid with elevated copy number (arbitrarily set to 1). The values are the average of at least two biological replicas. *E. coli* RV308 was used as host.

As for any expression system individual proteins are expressed at quite varying levels from *Pm,* and β-lactamase is not among the highly expressed proteins. In the original identification of the X, P and U variants the *bla* gene was used as a reporter and it is therefore of interest to study if the variant combinations would also stimulate the expression of genes other than *bla*. We selected the bacterial *celB* gene (encoding phosphoglucomutase) and the human *gm-csf* gene (encoding cytokine granulocyte-macrophage colony-stimulating factor) as representative examples for such an analysis. Both of these genes were previously (in contrast to *bla*) shown to be efficiently expressed from wild-type *xylS/Pm*. CelB was earlier found to be the clearly dominating protein on a crude gel when expressed from wild-type *xylS/Pm*[21] and also GM-CSF could be visualized on a protein gel when expressed from a plasmid with elevated copy number [14]. The *bla* gene in the constructs described above was therefore substituted with either the *celB* or the *gm-csf* gene. Phosphoglucomutase enzyme activities were then measured, while GM-CSF protein levels were visualized by Western Blotting (Figures 4a and 5a). In case of *gm-csf* another strain (RV308) was used as host to enable comparison of expression levels to previously published results [14]. Comparisons of ampicillin tolerances and analyses of *gm-csf* expression at the transcript and protein levels in DH5α andRV308 indicated that absolute values are slightly higher in RV308, while all relative values, compared to wild-type, were similar in both strains (data not shown).

Interestingly, expression of both genes was quite significantly stimulated at the protein level, particularly with the comXPU construct (about 8.5-fold for *celB*). For *gm-csf* protein production was difficult to detect by Western blotting, but we have shown before that it is easily detectable if a copy up (3–4 fold) version of the same plasmid is used [14]. Compared to this construct the comXPU variant (at wild-type plasmid copy number) produced between 3 and 4-fold more GM-CSF. This means that the level of stimulation per gene copy is probably around ten-fold and the protein was now clearly visible on a crude gel, without the need for Western Blotting.

We also monitored the stimulation at the transcript level, and interestingly, for *celB* the comXPU construct stimulated accumulation of target gene transcripts more (about 15-fold) than at the protein level (Figure 4b). This might be interpreted to indicate inclusion body formation but we have found that this particular protein does not to any significant extent form inclusion bodies (data not shown). We therefore conclude that under the reported conditions (with comXPU) the excessive transcript amounts cannot be efficiently handled by the translational machinery. For *gm-csf* the transcriptional stimulation was also highest for comXPU (4.8-fold), compared to the wild-type plasmid with wild-type copy number (Figure 5b).

Based on all these studies we conclude that the variant expression control elements isolated in the context of the *bla* gene also are able to stimulate the expression of other and very unrelated genes, although not to the same extent. The reason for this difference may either be that the variant expression elements are particularly well adapted to *bla*, or that the more efficient expression of *celB* and *gm-csf* in the wild-type system gives less room for further improvements.

### Chromosomal integration of genes may be a future option for optimized expression cassettes

Ideally chromosomal integrations can be seen as desirable in protein expression, due to greater genetic stability of the production strains. We have studied this for *bla*, *celB* and *gm-csf*, using the expression cassette with the comXPU variant, integrated into the *E. coli* chromosome (strain DH5α for *bla*, *celB,* and strain RV308 for *gm-csf*) by use of conditional-replication, integration and modular (CRIM) plasmids [22]. These replicons facilitate directed integration of a DNA-fragment at different locations in *E. coli* chromosome at bacterial attachment sites *(attB).* The *xylS/Pm* expression cassette with the three target genes was PCR-amplified and cloned into the integration plasmids, and successful insertion of single copies was confirmed by a PCR procedure described by Haldimann and Wanner [22]. Expression levels of the resulting strains were tested both by enzyme assays or Western Blots and qRT-PCR. For each gene the results were similar independent of the integration sites, and for β-lactamase the final protein expression was about 8-fold higher relative to the wild-type plasmid system (Figure 3b). For *celB* the corresponding levels were slightly lower than that of the wild-type cassette in the plasmid (Figure 4b), and for GM-CSF the protein was not detectable on a Western Blot (data not shown).

At the transcriptional level stimulation of *bla* expression was somewhat lower than at the translational level, as for XPU in the plasmid state. In contrast, for *celB* the transcription was much more stimulated in the plasmid state. For *gm-csf* accumulated transcript levels were about 5-fold lower than for the corresponding wild-type plasmid with wild-type copy-number (not shown).

In conclusion the results show that combination of optimized variant sequences in the expression control system lead to additive effects that can be used to strongly stimulate expression, but not sufficiently for single copy chromosomal integrations to reach the maximum expression levels obtained by the use of optimized plasmids. It may be possible to overcome this bottleneck by other means (see Discussion).

## Discussion

The wild-type *xylS/Pm* expression cassette has previously been found to express at least some proteins at industrial levels under high cell density conditions [13,14]. It is well known that no expression system can guarantee high levels of expression for any protein, and the reasons for this are obviously related to features of each specific gene or the protein encoded by it. It will in any case be important to use optimized inducible expression cassettes, particularly if the expression-stimulating features are acting on most genes. Most systems used for protein expression in *E. coli* are based on promoters that utilize the host RNA polymerase, with the exception of the bacteriophage T7 RNA polymerase based systems which appears to be prone to genetic instability [23].

From the results described here it is clear that the *xylS/Pm* cassette could be further improved relative to its wild-type version, and we believe that this potential has not yet been exhausted. It will clearly be important to reduce the enhanced uninduced expression level which was a consequence of the stimulated expression.

One important outcome of the studies reported here is that the improvement of the expression cassette seems to be valid at least to some extent for genes other than the one (*bla*) used to identify the variants. It appears likely that mutations stimulating transcription (X and P) might have a more universal effect than those stimulating translation (U), although we have found that sequences in the 5'-UTR region (or its corresponding DNA) may also affect transcription initiation rate [16]. The 5'-UTR may form secondary structures that potentially involve parts of the coding sequence of the gene to be expressed, but such problems can to some extent be predicted by bioinformatics analyses. It would be unrealistic to expect that all genes should respond quantitatively similar to changes in the sequences of the expression cassette, since the absolute expression levels vary over a wide range. This may explain most of the quantitative response differences observed among the genes tested in this study.

Chromosomal integration of genes to be expressed is an attractive approach in recombinant protein production, as it most likely will be associated with greater genetic stability. This approach appears to be possible for the T7 system if the growth conditions are modified and prolonged production times are considered acceptable [10], and also with a tandem used constitutive *tac* promoter system [12]. The study reported here indicates that under standard shake-flask conditions the integrated engineered XylS*/Pm* system can lead to enough transcript formation to allow expression levels comparable to those obtained by a plasmid-mediated system. Further improvement may be achieved by optimization of the growth conditions and we have also recently found (unpublished) that the level of expression is very sensitive to the intracellular concentration of the XylS regulator, a potential bottleneck that may be solved without the need for plasmids.

## Conclusions

Our results clearly show that previously identified stimulatory expression control elements for the inducible *xyls/Pm* cassette can be combined in one expression vector to further improve expression levels. We found this valid also for genes other than the one used to identify the variants in the first place. For *bla* this resulted in higher expression from a single chromosomally integrated gene copy than from the corresponding wild-type plasmid system. The approach used here should be applicable also to other expression cassettes and such optimized systems should represent a good starting point for the goal of producing recombinant proteins without the need for extra-chromosomal replicons.

## Methods

### Strains and growth conditions

*Escherichia coli* DH5α (Bethesda Research Laboratories) was used as a host for cloning and expression studies unless otherwise stated. Cells were grown in Luria-Bertani (LB) broth (10 g L^-1^ tryptone, 5 g L^-1^ yeast extract, and 5 g L^-1^ NaCl) or on LB agar (LB broth with 20 g L^-1^ agar) at 37°C, except for in expression studies, where 30°C was used. Kanamycin (50 μg μL^-1^) was used for selection purposes.

For expression studies with *gm-csf Escherichia coli* RV308 (ATCC 31608) was used as host to facilitate comparison with previously published results [14]. Cells were grown at 30°C in HiYe medium (8.6 g L^-1^ Na_2_HPO_4_·2H_2_O, 3 g L^-1^ KH_2_PO_4_, 1 g L^-1^ NH_4_Cl, 0.5 g L^-1^ NaCl, 2 g L^-1^ glucose, 10 g L^-1^ glycerol, 10 g L^-1^ yeast extract, 2.5 mM MgSO_4_, 250 μM Fe(III)-citrate, 49 μM H_3_Bo_3_, 79 μM MnCl_2_, 23 μM EDTA, 9 μM CuCl_2_, 10 μM Na_2_MoO_4_, 11 μM CoCl_2_, 36 μM Zn-acetate) and induced by addition of 0.5 mM m-toluate.

### Construction of plasmids and chromosomal integrations

The plasmid pTA16 was used as basis vector for construction of the combinations. pTA16 is a derivative of pIB11 [16], in which Agel and Sacl restriction sites were introduced at either end of the *xylS* gene. The *xylS* variants were obtained from the corresponding plasmids [17] upon digestion with Agel and Sacl. The *bla* gene was replaced by *celB* by the use of NdeI and BamHI restriction sites, and zby *gm-csf* by the use of NdeI and KpnI restriction sites. Agarose gel purifications were performed by QIAquick gel extraction kit (Qiagen). *Pm* and 5'-UTR variants were ordered synthetically (Eurofins MWG Operon, Germany) and were designed to carry overhangs suitable for cloning, Xbal/BspLUllI, and BspLUlll/Ndel, respectively. All constructs were confirmed by sequencing, performed by Eurofins MWG Operon (Germany).

The *xylS/Pm* expression cassette both with *bla, celB* and *gm-csf* as reporter gene was PCR-amplified with the primer set 5'-AAACACTAGTTCAGAGCTTGGAGAG-3' and 5'-CATAAAGCTGACTCTAGCTA-3' from plasmids pTA16_comXPU_bla, pTA16_comXPU_celBand pTA16_comXPU_gmcsf, respectively, and were cloned as BamHI/Spel-fragments (*bla, celB*) or KpnI/SpeI-fragments (*gm-csf*) into the integration plasmids. Chromosomal integration of the resulting plasmids was performed as described by Haldimann and Wanner [22].

The plasmids pAH63, pAH70 and pAH95 with the corresponding helper plasmids pINT-ts (*att*_*λ*_), pAH69 (*att*_HK022_) and pAH121 (*att*_P21_), respectively, were chosen for integration. Genomic location of the integration sites in the *E. coli* K-12 genome sequence are: for *att*_*λ*_ 806551, for *att*_HK022_ 1055412 and for *att*_P21_ 1210637 [24]. Successful integration could be confirmed for all three *bla*-constructs and for each two of the *celB*- and *gmcsf*-constructs. The integrants were named based on the pAH plasmids and the reporter genes used (Int_63_*bla*, Int_70_*bla*, Int_95_*bla*, Int_63_*celB*, Int_70_*celB*, Int_63_*gmcsf*, Int_70_*gmcsf*).

### Standard DNA manipulations

Transformations of *E. coli* were performed with a modified RbCl protocol (Promega) in cloning experiments. Wizard Plus SV mini preps DNA purification kit (Promega) was used for plasmid DNA purifications. Enzymatic manipulations were performed as described by the manufacturers. PCR reactions were performed using the Expand high fidelity PCR system kit (Roche).

### Enzyme assays

For enzyme assays growing strains were induced by addition of 0.1 mM m-toluate in exponential phase and samples were collected after four hours of continued growth at 30°C. Enzyme measurements (β-lactamase and phosphoglucomutase assay) were performed according to the procedures described previously [25,26]. All enzyme activity analyses were repeated at least twice, and measurements were carried out with minimum three technical recurrences.

### Western blotting

Cell samples were harvested by centrifugation (8000 rpm, 5 min, 4°C). Cell lysis was performed by resuspension of the pellets in 50 mM Tris–HCl, pH 8.0, addition of the same volume of sucrose solution (40% sucrose containing 2 mM EDTA in 50 mM Tris–HCl, pH 8.0), 125 UmL^-1^ Benzonase Nuclease and 0.5 mg mL^-1^ lysozyme and incubation at room temperature with shaking for 1 h. 3x sample buffer (150 mM Tris, pH 6.8, 30% glycerol, 6% SDS, 0.3% bromophenolblue, 300 mM DTT) was added, samples were separated by SDS-PAGE (17%, ClearPage, CBS Scientific) and transferred to a nitro-cellulose membrane. The membrane was blocked with Blotto and GM-CSF was detected by His-Probe-HRP in combination with ECL-substrate (Thermo Scientific) in Kodak Image Station 2000R (Kodak).

A plasmid with the wild-type expression cassette but containing the *trfA* variant *cop271C*[27] was used as positive control. This plasmid is maintained at a copy number 3.5 times higher than for plasmids with wild-type *trfA*.

### RNA isolation, cDNA synthesis and qRT-PCR

Strains were grown as described above (for enzyme assays). Cell cultures were stabilized with RNAprotect (Qiagen) prior to freezing and RNA was isolated from the frozen cell pellets using the RNAqeous kit (Ambion) as described by the manufacturers. The RNA preparations were treated with DNase (DNA-free, Ambion) and cDNA was produced from 3 mg total RNA as template using the First-Strand cDNA synthesis kit (Amersham Biosciences) with random pd(N)6 primers as described by the suppliers. Two-step qRT-PCR with the power SYBR green PCR master mix (Applied Biosystems) in a 7500 Real Time PCR System instrument (Applied Biosystems) was used for quantification of transcripts. PCR cycles were 95°C for 10 min, followed by 40 cycles of amplification (95°C for 15 s; 60°C for 1 min). Results were analysed using 7500 system software v1.3, and data were normalized by the 2^-ΔΔCT^ method [28]. Primers were designed using the primer express software (Applied Biosystems). Primer pairs used for transcript quantification were 5'-ACGTTTTCCAATGATGAGCACTT-3' and 5'-TGCCCGGCGTCAACAC-3' for *bla*, 5'-GTCCTCTTAGTTAAATGG-3' and 5'-AGGAATCGAACCTGC-3' for *celB* and 5'-CCCTGGGAGCATGTGAATG-3' and 5'-CATCTCAGCAGCAGTGTCTCTACTC-3' for *gm-csf.* 16S rRNA gene (primer pair 5'-ATTGACGTTACCCGCAGAAGAA-3' and 5'-GCTTGCACCCTCCGTATTACC-3') was used as a normalizer.

## Competing interests

The authors declare that they have no competing interests.

## Authors’ contributions

FZ and RL were involved in all aspects of the experimental design and execution. All authors contributed to the writing of the manuscript. All authors read and approved the final manuscript.
